# Is Replantation Associated With Better Hand Function After Traumatic Hand Amputation Than After Revision Amputation?

**DOI:** 10.1097/CORR.0000000000002906

**Published:** 2023-11-03

**Authors:** Joonas Pyörny, Patrick Luukinen, Ida Neergård Sletten, Aleksi Reito, Olli V. Leppänen, Jarkko Jokihaara

**Affiliations:** 1 Faculty of Medicine and Health Technology, Tampere University, Tampere, Finland; 2 Center for Musculoskeletal Diseases, Tampere University Hospital, Tampere, Finland; 3 Division of Orthopaedic Surgery, Oslo University Hospital, Oslo, Norway

## Abstract

**Background:**

Replantation is an established treatment for traumatic upper extremity amputation. Only a few studies, however, have assessed the patient-reported outcomes of replantation, and the findings of these studies have been conflicting.

**Questions/purposes:**

(1) Is replantation associated with better hand function than revision amputation? (2) Is replantation associated with better health-related quality of life, less painful cold intolerance, and more pleasing hand esthetics than revision amputation after a traumatic hand amputation?

**Methods:**

In this retrospective, comparative study, we collected the details of all patients who sustained a traumatic upper extremity amputation and were treated at the study hospital. Between 2009 and 2019, we treated 2250 patients, and we considered all patients who sustained a traumatic amputation of two or more digital rays or a thumb as potentially eligible. Based on that, 15% (334 of 2250) were eligible; a further 2% (8 of 334) were excluded because of a subsequent new traumatic amputation or bilateral amputation, and another 22% (72 of 334) refused participation, leaving 76% (254 of 334) for analysis here. The primary outcome was the DASH score. Secondary outcomes included health-related quality of life (EuroQOL-5D [EQ-5D-5L] Index), painful cold intolerance (the Cold Intolerance Symptom Severity score), and hand esthetics (the Michigan Hand Questionnaire aesthetic domain score). The minimum follow-up time for inclusion was 18 months. Patients were classified into two treatment groups: replantation (67% [171 of 254], including successful replantation in 84% [144 of 171] and partially successful replantation in 16% [27 of 171], in which some but not all of the replanted tissue survived), and revision (complete) amputation (33% [83 of 254], including primary revision amputation in 70% [58 of 83] and unsuccessful replantation followed by secondary amputation in 30% [25 of 83]). In this cohort, replantation was performed if possible, and the reason for choosing primary revision amputation over replantation was usually an amputated part that was too severely damaged (15% [39 of 254]) or was unattainable (2% [4 of 254]). Some patients (3% [8 of 254]) refused to undergo replantation, or their health status did not allow replantation surgery and postoperative rehabilitation (3% [7 of 254]). Gender, age (mean 48 ± 17 years in the replantation group versus 50 ± 23 years in the revision amputation group; p = 0.41), follow-up time (8 ± 4 years in the replantation group versus 7 ± 4 years in the revision amputation group; p = 0.18), amputation of the dominant hand, smoking, extent of tissue loss, or presence of arterial hypertension did not differ between the groups. Patients in the replantation group less frequently had diabetes mellitus (5% [8 of 171] versus 12% [10 of 83]; p = 0.03) and dyslipidemia (4% [7 of 171] versus 11% [9 of 83]; p = 0.04) than those in the revision group and more often had cut-type injuries (75% [129 of 171] versus 60% [50 of 83]; p = 0.02).

**Results:**

After controlling for potential confounding variables such as age, injury type, extent of tissue loss before treatment, and accident of the dominant hand, replantation was not associated with better DASH scores than revision amputation (OR 0.82 [95% confidence interval (CI) 0.50 to 1.33]; p = 0.42). After controlling for potential cofounding variables, replantation was not associated with better EQ-5D-5L Index scores (OR 0.93 [95% CI 0.56 to 1.55]; p = 0.55), differences in Cold Intolerance Symptom Severity scores (OR 0.85 [95% CI 0.51 to 1.44]; p = 0.79), or superior Michigan Hand Questionnaire esthetic domain scores (OR 0.73 [95% CI 0.43 to 1.26]; p = 0.26) compared with revision amputation.

**Conclusion:**

Replantation surgery was conducted, if feasible, in a homogenous cohort of patients who underwent amputation. If the amputated tissue was too severely damaged or replantation surgery was unsuccessful, the treatment resulted in revision (complete) amputation, which was not associated with worse patient-reported outcomes than successful replantation. These results contradict the assumed benefits of replantation surgery and indicate the need for credible evidence to better guide the care of these patients.

**Level of Evidence:**

Level III, therapeutic study.

## Introduction

Traumatic upper extremity amputations can be treated with a surgical revision (that is, surgical completion of the amputation, with or without primary closure) or replantation, which restores the vitality of the amputated tissue [33]. Typical indications for replantation in adults are the amputation of two or more digits, amputation of the thumb, and amputation proximal to the metacarpophalangeal joint [36]. In past decades, replantation surgery has become an established practice in many trauma centers [35].

Outcomes after replantation surgery have usually been reported using objective technical measures, such as survival of revascularized tissue, joint ROM, grip force, or test results for skin sensation. In general, revitalization of amputated tissue has been achieved in more than 80% of replanted digits [11, 32, 38, 39], but the function of the replanted digits rarely recovers completely [6]. Although patient-reported outcome measures (PROMs) are generally more relevant than objective outcome measures when assessing disability [15, 16, 29, 34, 45, 46], only a few studies on outcomes after replantation surgery have reported any PROM data [8, 10, 12, 21, 37, 43, 44, 48, 49], and the results of these studies have been conflicting. Overall, there is a lack of evidence about the benefits of replantation surgery [8, 11-13, 18, 19, 21, 22, 28, 38, 43, 44, 47-49]. There is also a scarcity of PROM data after revision amputation, and the data that are available suggest that minor disability occurs [8, 12, 16, 21, 43, 44, 48, 49]. Other studies reported fewer days of hospitalization, a lower number of secondary operations, and more rapid return to work compared with replantation surgery [5, 12, 21, 43, 48]. We therefore wished to evaluate the outcomes of replantation and revision amputation after traumatic distal upper extremity amputation.

In this study, we asked: (1) Is replantation associated with better hand function than revision amputation? (2) Is replantation associated with better health-related quality of life, less painful cold intolerance, and more pleasing hand esthetics than revision amputation after a traumatic hand amputation?

## Patients and Methods

### Study Design and Setting

This was a retrospective, comparative study performed at Tampere University Hospital, Tampere, Finland, a secondary and tertiary referral hospital serving a referral population of approximately 3 million people.

### Patients

**Fig. 1:**
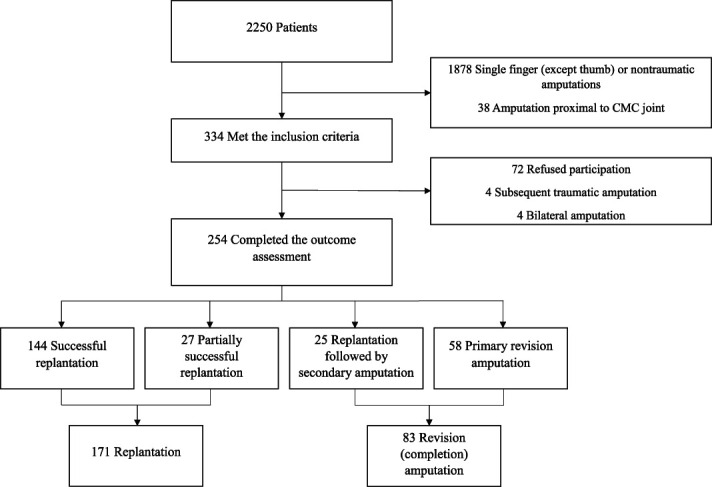
This flowchart demonstrates the patient selection for this study. CMC = carpometacarpal.

We screened all patients who had sustained a traumatic upper extremity amputation between 2009 and 2019 using the electronic medical records of the participating center. During the study period, we identified 2250 patients with traumatic upper extremity amputation. Of those, we considered all patients who sustained a traumatic amputation of two or more digital rays or a thumb as potentially eligible. Based on that, 15% (334 of 2250) were eligible; a further 2% (8 of 334) were excluded because they had a subsequent new traumatic amputation or bilateral amputation, and another 22% (72 of 334) refused participation, leaving 76% (254 of 334) for analysis here (Fig. 1). Patients were identified with diagnostic and treatment codes (Supplemental Tables 1 and 2; http://links.lww.com/CORR/B260). Replantation was conducted under the universal health coverage for all amputation injuries of two or more digits or amputation of the thumb, unless the amputated tissue was too severely damaged or missing [36]. The indications for replantation did not change during the study period.

For the study cohort, we included all injuries that had caused a fracture and loss of circulation in two or more digits proximal to the distal interphalangeal joint or in the thumb proximal to the interphalangeal joint. Injuries with some soft tissue in continuity were included. The exclusion criteria were amputation at or proximal to the carpometacarpal joint, bilateral amputations, a subsequent new traumatic amputation, or less than 18 months of follow-up. The minimum follow-up time was based on amputation studies with PROM assessments and was used to ensure that recovery had occurred [8, 43, 44, 48].

Patients were classified into two groups based on the treatment. The replantation group included successful replantation and partially successful replantation (some but not all replanted tissue survived). The revision (completion) amputation group included patients who underwent a primary revision amputation because replantation was not possible, as well as patients whose replantation was unsuccessful and in whom a subsequent separate secondary revision (completion) amputation of all replanted tissue was necessary. The reason that precluded a replantation was usually an amputated part that was too severely damaged (15% [39 of 254] or unattainable (2% [4 of 254]). Some patients refused the replantation (3% [8 of 254]) or their health status did not allow replantation surgery and postoperative rehabilitation (3% [7 of 254]).

### Patients

We collected patient, injury, and treatment details from the medical records. These included patient age, gender, hand dominance, smoking, and the presence of diabetes mellitus, hypertension, and dyslipidemia. Injury and treatment details included the amputation mechanism (cut, crush, or avulsion), initial and final extent of tissue loss (amputation level), primary and all secondary operations, major complications, and accident type (leisure or occupational). Major complications included pulmonary embolism, a hemodynamic condition that needed intensive care, or deep infection.

Gender, age (mean 48 ± 17 years in the replantation group versus 50 ± 23 years in the revision amputation group; p = 0.41), follow-up (8 ± 4 years in the replantation group versus 7 ± 4 years in the revision amputation group; p = 0.18), amputation of the dominant hand, smoking, extent of tissue loss, or presence of arterial hypertension did not differ between the groups (Table 1). Patients in the replantation group less frequently had diabetes mellitus (5% [8 of 171] versus 12% [10 of 83]; p = 0.03) and dyslipidemia (4% [7 of 171] versus 11% [9 of 83]; p = 0.04) than those in the revision group and more often had cut-type injuries (75% [129 of 171] versus 60% [50 of 83]; p = 0.02). Patient age ranged from 1 to 85 years at the time of injury, and 12 patients were younger than 18 years at the time of the assessment. Differences in accident types and presence of diabetes mellitus and dyslipidemia were not considered disqualifying problems because these did not affect treatment choice, and accident type was considered a potential confounding variable in the regression analysis.

**Table 1.** Demographic characteristics of the treatment groups

	Replantation (n = 171)^a^	Revision amputation (n = 83)^b^	p value
Age in years	48 ± 17	50 ± 23	0.41
Follow-up in years	8 ± 4	7 ± 4	0.18
Female	14 (24)	12 (10)	0.66
Dominant hand involved	53 (90)	48 (40)	0.51
Mechanism of injury			
Cut	75 (129)	60 (50)	0.02
Crush	11 (19)	23 (19)	
Avulsion and others	13 (23)	17 (14)	
Occupational accident	26 (45)	34 (28)	0.22
Smoking	27 (46)	19 (16)	0.19
Comorbidities			
Dyslipidemia	4 (7)	11 (9)	0.04
Diabetes mellitus	5 (8)	12 (10)	0.03
Arterial hypertension	20 (35)	27 (22)	0.28
Amputation groups			
Thumb-only-amputation	25 (43)	30 (25)	0.10
Two digits including thumb	6 (11)	4 (3)	
Two digits excluding thumb	25 (43)	40 (33)	
Three digits	23 (40)	14 (12)	
Four digits	15 (25)	10 (8)	
Five digits	5 (9)	2 (2)	

Data presented as mean ± (SD) or % (n).

a The replantation group includes patients who were successfully or partially successfully treated with replantation. The successful replantation subgroup contains patients who underwent a replantation that resulted in the survival of all replanted tissue. The partially successful replantation subgroup includes patients who received a replantation but not all the replanted tissue had sustained viability.

b The revision amputation group includes patients who were treated with primary revision (completion) amputation or with replantation followed by secondary amputation. The primary revision (completion) amputation subgroup includes patients who underwent a primary revision (completion) amputation without replantation. Replantation followed by secondary amputation includes patients who underwent an unsuccessful replantation attempt, which was followed by a subsequent separate secondary revision amputation.

The replantation success proportion was 73% (144 of 196) or 87% (171 of 196) if partially successful replantations were included. The extent of tissue loss did not differ between the two treatment groups before treatment, but it was smaller after replantation (Table 2). The correlation between the extent of tissue loss before treatment and the amount of successfully replanted tissue was r = 0.76 (95% confidence interval [CI] 0.69 to 0.82; p < 0.001). Five patients reported prosthesis use, and six patients underwent toe transfers (Supplemental Table 3; http://links.lww.com/CORR/B260).

**Table 2.** The extent of tissue loss, defined as the total number of functional joints lost before and after treatment in both groups

Amputation group	Replantation (n = 171)	Revision amputation (n = 83)	p value
All amputations			
Before treatment	4 (2-6)	3 (2-5)	0.02
After treatment	0 (0-2)	3 (2-5)	< 0.001
Thumb-only amputation			
Before treatment	1 (1-1)	1 (1-1)	0.94
After treatment	0 (0-0)	1 (1-1)	< 0.001
Two digits including thumb			
Before treatment	5 (3-5)	4 (4-6)	0.87
After treatment	2 (0-3)	4 (4-6)	0.02
Two digits excluding thumb			
Before treatment	4 (3-4)	3 (2-4)	0.28
After treatment	0 (0-2)	3 (2-4)	< 0.001
Three digits			
Before treatment	6 (5-7)	6 (4-6)	0.51
After treatment	2 (1-3)	6 (4-6)	< 0.001
Four digits			
Before treatment	8 (7-12)	9 (8-12)	0.48
After treatment	2 (0-5)	9 (8-12)	< 0.001
Five digits			
Before treatment	14 (12-14)	15 (14-15)	0.21
After treatment	5 (2-8)	15 (14-15)	0.03

Data presented as median (IQR).

### Surgical Techniques and Aftercare

Replantation surgery was performed by specialized hand surgeons in an emergency operation. A continuous brachial plexus block was used for anesthesia, and it was continued for 5 days postoperatively. The standard operating technique consisted of blood vessel and nerve anastomosis or reconstruction under microscope magnification. Postoperative monitoring in the ward was continued for 5 to 7 days, and intensive outpatient rehabilitation was conducted for least 3 to 6 months [36]. In a primary revision (completion) amputation, unviable injured tissue was debrided, and the remaining soft tissue defect was covered with direct sutures or reconstructed with a flap. In a secondary revision (completion) amputation, all unviable tissue was excised, and the remaining tissue defect was covered in a separate operation after an unsuccessful replantation.

### Primary and Secondary Study Outcomes

The primary outcome was the DASH [23, 25], which is a validated instrument for assessing upper extremity function and symptoms that correlates well with functional physical tests after an amputation [16]. DASH grades the upper limb disability on a 0 to 100 scale, where 0 represents perfect function and no pain [23]. For patients younger than 18 years, we used the QuickDASH. This version includes only a subset of questions that are more suitable and better validated for children [2, 30]. The QuickDASH score is scaled similarly to the full DASH score [20]. The normative DASH score for the general population varies between 5 and 20 points for persons aged between 20 and 70 years [1, 17, 24, 31]. The minimum clinically important difference for the DASH is estimated to be 10 (95% CI 7 to 14) [20]. Accordingly, we used a cutoff limit of 20 points for the DASH to identify patients with reduced upper extremity function.

Our secondary outcomes were health-related quality of life as measured by the EuroQOL-5 Dimensions (EQ-5D-5L) [41], Cold Intolerance Symptom Severity (CISS) score [26], Michigan Hand Questionnaire (MHQ) [7] esthetics domain, return to work, and use of hand prostheses, as well as a question on a numerical rating scale ranging from 0 to 10: “How much did the appearance of your hand bother you during the previous week?” (0 = very much, 10 = not at all). For the EQ-5D-5L, local population parameters were not available; therefore, to calculate the index value, we used values from the Danish population, which is culturally and socioeconomically similar to the Finnish population [27]. With the CISS, scores of more than 50 points were defined as abnormal cold sensitivity [4, 42]. To avoid redundant assessment of hand function, we included only the esthetic domain of the MHQ on a scale from 0 (worst) to 100 (best).

For the multivariate analysis, we quantified the extent of (amputated) tissue loss on an ordinal scale by determining the number of lost joints; for example, amputation of two digits at the level of the proximal phalanx equaled four lost joints. For the supplementary bivariate analyses, the extent of the amputation was described with six categories: thumb only, two digits including the thumb, two digits excluding the thumb, three digits, four digits, and five digits. In these categories, we used the extent of tissue loss to further describe the baseline (before treatment) and how much tissue was successfully replanted.

### Ethical Approval

Ethical approval for this study was obtained from Tampere University Hospital, Tampere, Finland. The study is reported in accordance with the STROBE guidelines [14].

### Statistical Analysis

We estimated the association of the independent baseline variables for the outcomes using ordinal regression. For the multivariate analysis, we included patient age, sex, injury type (cut, crush, or avulsion), intervention (replantation or revision amputation), the extent of tissue loss before treatment, and whether the injured hand was the dominant hand as independent variables based on previous studies [6, 44] and clinical experience. Overall r^2^ coefficients, which were used to interpret the applicability of baseline variables, and p values are reported. We used the Wald test to evaluate the association between the independent variables and dependent outcome variable. ORs of the treatment variable are reported as revision amputation compared with replantation (revision amputation/replantation). We used restricted cubic splines with four knots to model the relationship between age and outcome in regression analyses, leading to three different ORs for the age variable (age 1, age 2, and age 3). Assumptions of ordinal regression were analyzed and estimated from the data.

In a supplementary analysis, we present continuous outcomes as medians and IQRs, and used the Wilcoxon rank sum test to compare continuous outcomes between the two treatment groups. Patient characteristics are presented as the mean and SD in case of normal distribution, and we used Welch t-test for comparison. We used the chi-square test or the Fisher exact test to compare categorical variables. We did not calculate p values in the subgroup analysis because of small sample sizes.

We measured the association between two quantitative variables using Spearman correlations, and the CIs were calculated via Z transformation. We considered correlations of 0 to 0.19 as very weak, 0.20 to 0.39 as weak, 0.40 to 0.59 as moderate, 0.60 to 0.79 as strong, and 0.80 to 1 as very strong. A p value less than 0.05 was considered statistically significant. All analyses were conducted using R version 4.2 (R Foundation for Statistical Computing).

We performed a power analysis after data collection. We used previous studies of upper extremity amputations to estimate variation in DASH scores [8, 12, 21, 43, 44, 48]. With a difference of 10 ± 15 points for the DASH score between treatment groups and 80% power to perceive a difference between the replantation and revision amputation groups (two-sided test with alpha value 0.05), the sample size was estimated to be 35 patients in each treatment group.

## Results

### Association Between Treatment (Replantation or Amputation) and DASH Score

**Fig. 2:**
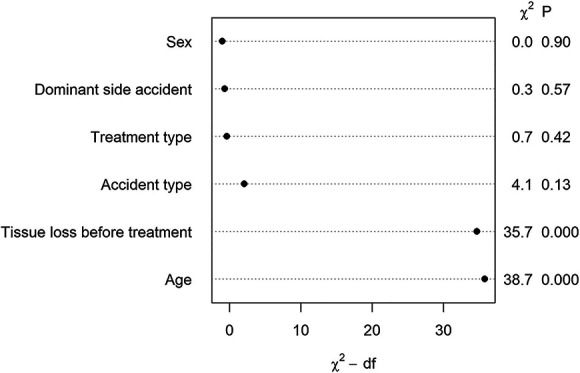
Ordinal regression analysis shows the association of patient and injury characteristics for the DASH. The Wald test describes the relative importance of the association of the independent variables (the greater number indicates superior importance). Overall r^2^ coefficient = 0.255.

After controlling for potential confounding variables such as accident type, extent of tissue loss before treatment, and accident of the dominance hand, replantation was not associated with better DASH scores than revision amputation (OR 0.82 [95% CI 0.50 to 1.33]; p = 0.42). We found that patient age (age 1 OR 1.10 [95% CI 1.05 to 1.14]; p < 0.001, age 2 OR 0.89 [95% CI 0.83 to 0.96]; p = 0.003, and age 3 OR 2.38 [95% CI 1.30 to 4.36]; p = 0.005) and extent of tissue loss before treatment (OR 2.31 [95% CI 1.76 to 3.04]; p < 0.001) were associated with DASH scores (Fig. 2). There was no difference in DASH scores between the treatment groups (Supplemental Table 4; http://links.lww.com/CORR/B260).

### Health-related Quality of Life, Cold Intolerance, and Esthetics

**Fig. 3:**
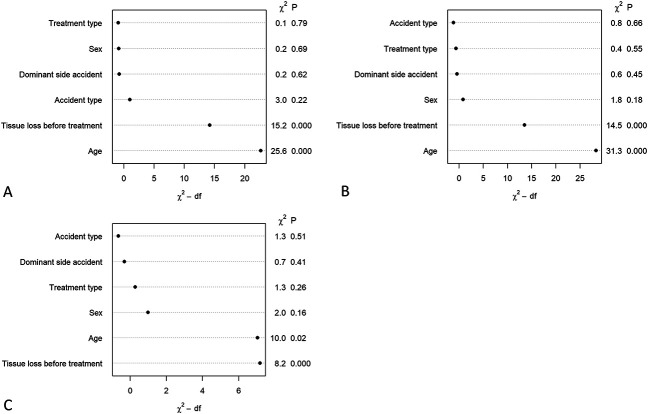
Ordinal regression analysis demonstrates the association of patient and injury characteristics for (**A**) the EQ-5D-5L index, (**B**) the CISS, and (**C**) the MHQ esthetic domain. The Wald test describes the relative importance of the association of the independent variables (the greater number indicates superior importance). The overall r^2^ coefficients were 0.149 for the EQ-5D-5L index model, 0.204 for the CISS model, and 0.096 for the MHQ esthetic domain model.

After controlling for potential cofounding variables, replantation was not associated with better EQ-5D-5L Index scores (OR 0.93 [95% CI 0.56 to 1.55 ]; p = 0.55), differences in CISS scores (OR 0.85 [95% CI 0.51 to 1.44]; p = 0.79), or superior MHQ esthetic domain scores (OR 0.73 [95% CI 0.43 to 1.26 ]; p = 0.26) than revision amputation. We found that patient age and extent of tissue loss before treatment were associated with differences in EQ-5D-5L Index scores (Fig. 3A), CISS scores (Fig. 3B), and MHQ aesthetic domain scores (Fig. 3C).

There were no differences in health-related quality of life, cold intolerance, and esthetics between the replantation and revision amputation groups when considering injury level (Supplemental Table 5; http://links.lww.com/CORR/B260^)^. The correlations between DASH scores and secondary outcomes were moderate or strong (Table 3).

**Table 3.** Correlations between different outcome variables and total DASH score

	r coefficient (95% CI)	p value
EQ-5D index	-0.72 (-0.78 to -0.66)	< 0.001
EQ-VAS	-0.55 (-0.64 to -0.46)	< 0.001
CISS	0.72 (0.65 to 0.78)	< 0.001
MHQ esthetics	-0.42 (-0.53 to -0.30)	< 0.001
Hand esthetics interference	-0.45 (-0.55 to -0.35)	< 0.001
Tissue loss before treatment	0.32 (0.20 to 0.43)	< 0.001
Tissue loss after treatment	0.17 (0.05 to 0.29)	0.007

The DASH is scored from 0 to 100, where 0 indicates no disability; the EQ-5D is scored from 0 to 1, where 1 indicates the best situation; the EQ VAS, or the EQ-5D-5L health state with VAS value, is scored from 0 to 100, where 100 indicates the best situation; the CISS is scored from 0 to 100, where 0 indicates no symptoms; the MHQ esthetics is scored from 0 to 100, where 100 indicates the best situation for hand esthetics interference (“How much did the appearance of your hand bother you during the previous week?”, 0 to 10, where 10 indicates “not at all”); tissue loss before treatment is defined as the quantitative variable of the number of lost joints before treatment; tissue loss after treatment is defined as the quantitative variable of the number of lost joints after treatment.

## Discussion

Replantation surgery has become the established treatment of upper extremity amputation injuries during the past four decades. Nevertheless, there is still lack of patient-rated treatment outcomes; therefore, replantation surgery could become an established practice based on its assumed benefits. In this study, we evaluated PROMs in a homogenous group of patients with upper extremity amputation treated with replantation or revision (completion) amputation, and we did not find associations between replantation and more favorable patient-rated outcomes related to hand function, disability, health-related quality of life, cold intolerance, or esthetics compared with revision (completion) amputation. Furthermore, the patient-rated outcomes of replantation were not poor; most patients reported no or minor disability, and this was similar in patients who underwent revision amputation. These findings suggest we should consider more stringent indications for replantation surgery; thus, we need comparative data on the outcomes that are the most important to the patients.

Cook et al. [9] described the reasons why different interventions may demonstrate similar outcomes. First, the outcome may bias findings; therefore, we used a patient-rated assessment that emphasizes outcomes that are important to patients [15, 16, 46] and can be used after an amputation injury, regardless of different treatment types. In contrast, measurements of skin sensation, joint movement, or grip force are misleading if the pertinent structures have been lost in one of the treatment groups, whereas a patient-rated assessment of hand function includes these functional deficiencies on a meaningful scale. A second reason for the lack of benefit could be poor treatment fidelity [9]. The proportion of successful replantations in our cohort was similar to that reported in previous studies [11, 32, 38, 39], and there was a strong correlation between the extent of tissue loss before treatment and the amount of successfully replanted tissue. Other possible explanations for similar outcomes after intuitively different treatments are shared mechanisms and nonspecific common factors that affect the outcome, regardless of the intervention [9]. Although context effects (influence of, for example, patient-physician relationship, treatment characteristics, health care setting, and patient expectations) may influence patient-reported outcomes [3], in our study, they probably fail to explain the lack of differences in outcomes, particularly because context effects might favor replantation, which is more-comprehensive care.

### Limitations

The lack of random allocation into treatment groups is a major limitation of our study. A patient characteristic such as a comorbidity or surgeon preference may have influenced the treatment decision and led to selection bias. However, without the results of the present study, it is difficult to warrant a randomized trial because replantation surgery is an established practice, despite the lack of credible evidence [8, 12, 43, 48, 49]. In our study, we found no evidence of selection bias; patients and injuries were generally similar in both groups (see Table 1), and factors that most likely influence the treatment decision (patient age, sex, injury type, the extent of tissue loss, and whether the injured hand was the dominant hand) were included in the primary multivariate analysis as potential confounding variables. The most common reason for choosing primary revision amputation over replantation was an amputated part that was too severely damaged or unattainable. Replantation was always performed, if possible (that is, if amputated tissue was viable), and health status influenced the treatment decision in only 3% of patients. Patients in the revision (completion) amputation group more often had dyslipidemia and diabetes, but these were not seen as disqualifying factors because they did not affect the treatment allocation. However, a vascular condition such as dyslipidemia or diabetes may have decreased the proportion of successful replantations and thus predisposed patients to eventual revision (completion) amputation.

It is possible that the response rate was lower in more-symptomatic patients, which would cause transfer bias. We think this bias is unlikely to be substantial because the patient details and injury characteristics were similarly distributed among responders and nonresponders. In our experience, more-symptomatic patients usually actively contact the replantation center for various reasons, and overall the response rate was high (76%). The use of PROMs as primary and secondary outcome variables minimizes the assessment bias. However, the absence of an association between replantation and its assumed benefits in our study might have been because the outcome variables were not sensitive or broad enough. Our primary outcome, DASH, is a general well-documented instrument that has been used to evaluate the outcome of upper extremity amputation [10, 16, 37, 40, 46]. It does not necessarily capture all issues related to patients with distal amputations and may have a floor effect for distal hand disability. However, it correlates well with functional hand tests [16], and 28 of 30 items in DASH reflect distal hand disability. In our study, the strong correlation between DASH and the secondary outcomes suggests that the DASH score meaningfully reflected the treatment outcome. Secondary outcomes were selected to cover all the essential domains that are affected by an amputation injury. Because of a relatively small number of certain amputation types, we might have missed some uncommon problems, and it also prevented us from comparing treatment outcomes in more specific subgroups. Based on the post hoc power analysis, our cohort size was acceptable. Another limitation of our study was its single-center study design, which limits the generalizability of the results. The study center is a specialized hand surgery unit that is the centralized and only provider of replantation for the referral area, and all operations are covered under universal healthcare.

### Association Between Treatment (Replantation or Amputation) and DASH Score

We found that replantation was not associated with better DASH scores than revision (completion) amputation. The finding of the absence of benefits from replantation surgery was not because of the unsatisfactory results of replantation but rather because these was no or minor disability after revision (completion) amputation. Based on this, surgeons might consider more stringent indications for replantation surgery. In previous studies that reported PROMs after an upper extremity amputation, the results of replantation have been inconsistent [8, 10, 12, 21, 37, 43, 44, 48, 49]. According to data from the United States and Asia [8], the outcome of replantation was better than revision amputation when three or more digits (including the thumb) were amputated if the difference was controlled for in the propensity score; there was no benefit from replantation of a thumb, two digits (regardless of whether the thumb was involved), or three or more digits if the thumb was not amputated. A recent systematic review and meta-analysis [43] of single-digit amputations suggested better outcomes after thumb replantation, whereas in other single-finger amputations, the difference was likely too small to be clinically meaningful. However, the review rated the evidence as low-quality, and the data were geographically unilaterally distributed [43].

### Health-related Quality of Life, Cold Intolerance, and Esthetics

Likewise, replantation was not associated with higher EQ-5D-5L scores, differences in cold intolerance, or esthetics than completion amputation. Based on this, we think disability after an amputation and the assumed benefits of replantation should be studied further with variables that are the most important to patients to provide proper information for decision-making about the treatment of amputation injuries. In our study, two-thirds of patients had CISS scores within the normative values after both treatment types. Similar data have also been reported in a previous study on replantation [37].

### Conclusion

Our study failed to show any benefit of replantation of an amputated thumb or two or more digits. Some patients with certain types of digital amputation, such as very proximal thumb amputations, might benefit from replantation. A randomized trial has been considered an unethical research method in upper extremity amputation treatment [47], but our results and data from other studies [8, 11-13, 19, 21, 22, 28, 38, 43, 44, 47-49] suggest that a randomized trial is warranted. The current indications for distal replantation surgery in many hand surgery units are mostly based on the survival potential of the amputated parts because surgeons and patients assume the functional and esthetic benefit of replantation instead of revision (completion) amputation surgery. However, unless a proper trial can provide a credible estimate of the treatment effect in at least some amputation types, performing resource-demanding distal replantation surgery in the absence of any evidence of its efficacy and effectiveness cannot be justified.

## Supplementary Material
