# Supplementary material for: Is Replantation Associated With Better Hand Function After Traumatic Hand Amputation Than After Revision Amputation?
Source: Clin Orthop Relat Res. 2023 Nov 3;482(5):843–53. doi: 10.1097/CORR.0000000000002906 (PMC11008649; doi:10.1097/CORR.0000000000002906)
Supplement: Supplementary file 1 [file abjs-482-843-s001.docx]

**Supplemental Table 1.** ICD-10 codes that were used to identify patients with traumatic upper extremity amputation

| **ICD 10 code** | **Diagnosis** |
| --- | --- |
| S47 | Crushing injury of shoulder and upper arm |
| S48.0 | Traumatic amputation at shoulder joint |
| S48.1 | Traumatic amputation at level between shoulder and elbow |
| S48.9 | Traumatic amputation of shoulder and upper arm, level unspecified |
| S57.0 | Crushing injury of elbow and forearm |
| S57.8 | Crushing injury of other parts of forearm |
| S57.9 | Crushing injury of forearm, part unspecified |
| S58.0 | Traumatic amputation at elbow level |
| S58.1 | Traumatic amputation at level between elbow and wrist |
| S58.9 | Traumatic amputation of forearm, level unspecified |
| S67.0 | Crushing injury of thumb and other finger(s) |
| S67.8 | Crushing injury of other and unspecified parts of wrist and hand |
| S68.0 | Traumatic amputation of thumb (complete)(partial) |
| S68.1 | Traumatic amputation of other single finger (complete)(partial) |
| S68.2 | Traumatic amputation of two or more fingers alone (complete)(partial) |
| S68.3 | Combined traumatic amputation of (part of) finger(s) with other parts of wrist and hand |
| S68.4 | Traumatic amputation of hand at wrist level |
| S68.8 | Traumatic amputation of other parts of wrist and hand |
| S68.9 | Traumatic amputation of wrist and hand, level unspecified |

**Supplemental Table 2**. Nordic Classification of Surgical Procedures codes that were used to identify patients who received treatment for traumatic upper extremity amputation

| **NCSP code** | **Surgical procedure** |
| --- | --- |
| NDP10 | Replantation of hand |
| NDP12 | Replantation of a digit |
| NDP18 | Replantation of several digits |
| NDP30 | Repair of wrist or hand by transplant of tissue |
| NDP32 | Repair of finger or fingers by transplant of tissue |
| NDQ10 | Amputation in wrist or hand |
| NDQ20 | Amputation of finger |
| NDQ48 | Revision or amputation or exarticulation of finger |
| NDQ60 | Revision of amputation or exarticulation stump in wrist |
| NCP10 | Replantation of forearm |
| NCP30 | Reconstruction of underarm with revascularization |
| NCQ10 | Exarticulation of elbow |
| NCQ20 | Amputation of forearm |
| NCQ48 | Revision of exarticulation or amputation stump of elbow or forearm |
| NBP10 | Replantation of upper extremity above elbow |
| NBQ10 | Humeroscapular exarticulation |
| NBQ20 | Amputation in upper arm |
| NBQ39 | Insertion of bone-anchored transdermal humeral implant |
| NBQ48 | Revision of amputation or exarticulation stump in shoulder or upper arm |
| NBQ49 | Insertion of humeral implant to give form to the amputation stump |
| PBC08 | Suture of axillary artery |
| PBC09 | Suture of brachial artery |
| PBC10 | Suture of radial artery |
| PBC11 | Suture of ulnar artery |
| PBC99 | Suture of other artery of upper extremity |

**Supplemental Table 3.** DASH score and information on patients who used a prosthesis and those who underwent toe transfers

| **Prosthesis user** | **Age at accident in years** | **Amputation level** | **Treatment type** | **DASH** |
| --- | --- | --- | --- | --- |
| Patient 1 | 65 | Three digits | Revision amputation | 0 |
| Patient 2 | 65 | Four digits | Revision amputation | 21 |
| Patient 3 | 51 | Five digits | Revision amputation | 17 |
| Patient 4 | 76 | Five digits | Partially successful replantation | 28 |
| Patient 5 | 16 | Five digits | Revision amputation | 40 |
| **Toe transfer** | **Age at accident in years** | **Amputation level** | **Treatment type** | **DASH** |
| Patient 1 | 49 | Four digits | Partially successful replantation | 41 |
| Patient 2 | 1 | Three digits | Revision amputation | 7 |
| Patient 3 | 42 | Thumb | Revision amputation | 13 |
| Patient 4 | 49 | Four digits | Partially successful replantation | 28 |
| Patient 5 | 18 | Thumb | Revision amputation | 2 |
| Patient 6 | 12 | Five digits | Replantation | 26 |

**Supplemental Table 4.** The number and percentage of patients with DASH scores of more than 20 points and CISS scores of more than 50 points for each amputation group

| **DASH score > 20 points** | **Replantation** | **Revision amputation** | **p value** |
| --- | --- | --- | --- |
| Thumb amputation | 28 (13) | 29 (6) |  |
| Two-digit amputation (including thumb) | 40 (4) | 0 (0) |  |
| Two-digit amputation (excluding thumb) | 22 (9) | 28 (9) |  |
| Three-digit amputation | 16 (40) | 17 (2) |  |
| Four-digit amputation | 58 (14) | 50 (4) |  |
| Five-digit amputation | 44 (6) | 50 (1) |  |
| All amputations | 37 (62) | 28 (22) | 0.12 |
| **CISS score > 50 points** | **Replantation** | **Revision amputation** | **p value** |
| Thumb amputation | 18 (7) | 21 (4) |  |
| Two-digit amputation (including thumb) | 60 (6) | 50 (1) |  |
| Two-digit amputation (excluding thumb) | 27 (9) | 20 (5) |  |
| Three-digit amputation | 47 (16) | 20 (2) |  |
| Four-digit amputation | 64 (14) | 38 (3) |  |
| Five-digit amputation | 17 (1) | 0 (0) |  |
| All amputations | 37 (53) | 23 (15) | 0.05 |

Data presented as % (n); the percentages are calculated in each treatment group for each amputation type; the p value represents the comparison between replantation and revision amputation.

**Supplemental Table 5.** Outcomes for each treatment group

|  | **Number** | **Replantation** | **Number** | **Revision amputation** | **p value** |
| --- | --- | --- | --- | --- | --- |
| Total | 171 |  | 83 |  |  |
| DASH | 167 | 13 (4-28) | 81 | 9 (3-21) | 0.19 |
| EQ-5D index | 167 | 0.8 (0.7-0.9) | 81 | 0.8 (0.7-1.0) | 0.78 |
| EQ VAS | 169 | 80 (70-90) | 82 | 80 (70-90) | 0.41 |
| CISS | 144 | 41 (24-60) | 65 | 37 (23-50) | 0.15 |
| MHQ aesthetics | 145 | 75 (56-94) | 66 | 72 (50-94) | 0.67 |
| Hand aesthetics interference | 170 | 9 (7-10) | 82 | 9 (8-10) | 0.76 |
| Thumb | 43 |  | 25 |  |  |
| DASH | 43 | 11 (2-22) | 24 | 5 (1-21) |  |
| EQ-5D index | 41 | 0.9 (0.8-1.0) | 24 | 0.8 (0.7-1.0) |  |
| EQ VAS | 42 | 81 (75-90) | 25 | 80 (60-90) |  |
| CISS | 39 | 28 (18-43) | 19 | 37 (20-50) |  |
| MHQ aesthetics | 39 | 92 (56-100) | 23 | 81 (62-100) |  |
| Hand aesthetics interference | 43 | 10 (8-10) | 25 | 9 (8-10) |  |
| Two digits including thumb | 11 |  | 3 |  |  |
| DASH | 10 | 16 (13-25) | 3 | 7 (4-13) |  |
| EQ-5D index | 11 | 0.8 (0.7-0.8) | 3 | 1.0 (0.9-1.0) |  |
| EQ VAS | 11 | 80 (70-90) | 3 | 83 (79-92) |  |
| CISS | 10 | 53 (47-62) | 2 | 51 (46-57) |  |
| MHQ aesthetics | 8 | 63 (39-72) | 2 | 72 (70-73) |  |
| Hand aesthetics interference | 11 | 10 (6-10) | 3 | 8 (5-9) |  |
| Two digits excluding thumb | 43 |  | 33 |  |  |
| DASH | 41 | 6 (1-20) | 32 | 10 (5-21) |  |
| EQ-5D index | 41 | 0.9 (0.8-1.0) | 32 | 0.8 (0.7-0.9) |  |
| EQ VAS | 42 | 80 (73-90) | 32 | 80 (67-91) |  |
| CISS | 33 | 32 (24-53) | 25 | 30 (20-45) |  |
| MHQ aesthetics | 37 | 81 (63-94) | 23 | 81 (50-94) |  |
| Hand aesthetics interference | 42 | 10 (8-10) | 32 | 9 (8-10) |  |
| Three digits | 40 |  | 12 |  |  |
| DASH | 40 | 14 (5-33) | 12 | 8 (3-17) |  |
| EQ-5D index | 40 | 0.8 (0.7-0.9) | 12 | 0.8 (0.8-1.0) |  |
| EQ VAS | 40 | 80 (70-86) | 12 | 88 (79-90) |  |
| CISS | 34 | 48 (32-66) | 10 | 40 (29-48) |  |
| MHQ aesthetics | 35 | 69 (47-91) | 9 | 69 (56-81) |  |
| Hand aesthetics interference | 40 | 9 (5-10) | 12 | 10 (9-10) |  |
| Four digits | 25 |  | 8 |  |  |
| DASH | 24 | 29 (11-45) | 8 | 18 (13-30) |  |
| EQ-5D index | 25 | 0.8 (0.7-0.9) | 8 | 0.8 (0.8-0.9) |  |
| EQ VAS | 25 | 75 (60-85) | 8 | 85 (80-90) |  |
| CISS | 22 | 60 (40-75) | 8 | 41 (33-64) |  |
| MHQ aesthetics | 18 | 63 (41-81) | 7 | 63 (47-94) |  |
| Hand aesthetics interference | 25 | 7 (5-10) | 8 | 10 (9-10) |  |
| Five digits | 9 |  | 2 |  |  |
| DASH | 9 | 28 (6-37) | 2 | 28 (23-34) |  |
| EQ-5D index | 9 | 0.8 (0.7-0.8) | 2 | 0.8 (0.8-0.8) |  |
| EQ VAS | 9 | 80 (50-80) | 2 | 77 (76-78) |  |
| CISS | 6 | 35 (20-49) | 1 | 47 (-) |  |
| MHQ aesthetics | 8 | 88 (66-100) | 2 | 38 (34-41) |  |
| Hand aesthetics interference | 9 | 8 (5-10) | 2 | 6 (5-6) |  |

Data presented as median (IQR); the p value represents the univariate comparison between replantation and revision amputation; the DASH is scored from 0 to 100, where 0 indicates no disability); the EQ-5D is scored from 0 to 1, where 1 indicates the best situation; the EQ VAS, or the EQ-5D-5L health state with VAS value, is scored from 0 to 100, where 100 indicates the best situation; the CISS is scored from 0 to 100, where 0 indicates no symptoms; the MHQ aesthetics is scored from 0 to 100, where 100 indicates the best situation for hand aesthetics interference (“How much did the appearance of your hand bother you during the previous week?”, 0 to 10, where 10 indicates “not at all”); tissue loss before treatment is defined as the quantitative variable of the number of lost joints before treatment; tissue loss after treatment is defined as the quantitative variable of the number of lost joints after treatment.
